# Assessing Health Inequalities in Iran: A Focus on the Distribution of Health Care Facilities

**DOI:** 10.5539/gjhs.v6n4p285

**Published:** 2014-05-07

**Authors:** Masoud Abolhallaje, Seyyed Meysam Mousavi, Mina Anjomshoa, Ali Beigi Nasiri, Hesam Seyedin, Jamil Sadeghifar, Aidin Aryankhesal, Ghasem Rajabi Vasokolaei, Mostafa Beigi Nasiri

**Affiliations:** 1 School of Health Management and Information Sciences, Iran University of Medical Sciences, Tehran, Iran; 2 Research Center for Modeling in Health, Institute for Futures Studies in Health, Kerman University of Medical Sciences, Kerman, Iran; 3 Students’ Scientific Research Center, Department of Health Management and Economics, School of Public Health, Tehran University of Medical Sciences, Tehran, Iran; 4 Research Center for Health Services Management, Institute for Futures Studies in Health, Kerman University of Medical Sciences, Kerman, Iran; 5 Graduated in MBA, Jawaharlal Nehru Technological University, Hyderabad, India; 6 Health Management and Economics Research Center, School of Health Management and Information Sciences, Iran University of Medical Sciences, Tehran, Iran; 7 Department of Health Education, School of Public Health, Ilam University of Medical Sciences, Ilam, Iran; 8 Hospital Management Research Center, Iran University of Medical Sciences, Tehran, Iran; 9 Shahid Chamran University, Ahvaz, Iran

**Keywords:** health inequality, distribution, health care, facility, Iran

## Abstract

**Background and objective:**

Equality in distribution of health care facilities is the main cause for access and enjoyment to the health. The aim of this study was to examine the regional disparities in health care facilities across the Markazi province.

**Methods:**

This was a cross-sectional study. Study sample included the cities of Markazi province, ranked based on 15 health indices. Data was collected by a data collection form made by the researcher using statistical yearbook. The indices were weighted using Shannon entropy. Finally, technique for order preference by similarity to ideal solution (TOPSIS) was used to rank the towns of the province in terms of access to health care facilities.

**Results:**

There is a large gap between cities of Markazi province in terms of access to health care facilities. Shannon entropy introduced the number of urban health centers per 1000 people as the most important indicator and the number of rural active health house per 1000 people as the less important indicator. According to TOPSIS, the towns of Ashtian and Shazand ranked the first and last (10^th^) respectively in access to health services.

**Conclusion:**

There are significant inequalities in distribution of health care facilities in Markazi province. We propose that policy makers determine resource allocation priorities according to the degree of development for a balanced and equal distribution of health care facilities.

## 1. Introduction

A balanced distribution of services and facilities is a step towards elimination of regional imbalances, because bigger regional differences in various respects move the population and capital to the poles (Shaykh Baygloo, 2012). To achieve human development and economic and sustainable development, it is necessary to minimize disparities, as any development; regardless of equality in society will be ultimately unstable (Zarrabi & Shaykh Baygloo, 2011). Various studies have been conducted with different attitudes on regional disparities in different geographic areas and identification of backward areas. The studies have been mostly conducted on the use of economic and social indices and infrastructure development indices to classify different geographical areas, in terms of the enjoyment of the indicators (Poor Fathi & Asheri, 2010).

Gross Domestic Product (GDP) and GDP per capita were the main indicators to assess development level. These indicators do not consider fairness in the distribution of health services and other social services (Mahmoudi, 2011). Health and development are closely linked to each other and can affect interchangeably (Rafi’iyaan & Taajdaar, 2008). Health sector as an important social part of any country plays a decisive role in the wellbeing of people (Sayemiri & Sayemiri, 2001).

Regional health inequalities are mainly a result of differences in the level of economic development and differences in access to health care facilities (Barakpuor, 2004). Access to health care is a multi-dimensional concept (Paez, Mercado, Farber, Morency, & Roorda, 2010). It is worth noting that before planning and performing the appropriate action to remove the inequities of “health”, planners and policy makers should be familiar with different parameters related to the quality and access to a variety of facilities and services in different regions of a country (Comber, Brunsdon, & Radburn, 2011; Zarrabi & Shaykh Baygloo, 2011). Because measuring the health indices is the most desirable and appropriate way to assess the level of health in a population (Sayemiri & Sayemiri, 2001). In fact, the analyzing of health indices and its distribution in different geographical areas is caused to recognize an imbalance in the distribution of health facilities and it also helps in planning for the equitable distribution and accessibility to services for all members of the society in an appropriate manner (Zarabi, Mohammadi, & Rakhshani Nasab, 2008). There are inequalities in access to health services both in developed and developing countries but it is more prevalent in developing countries (Zarrabi & Shaykh Baygloo, 2011). In developing countries, individuals’ quality of life is affected by the huge regional disparities which are rapidly increasing in many cases (Khakpour, 2007).

Health system in Iran like other countries is faced with growing changes in socio-economic dimensions and additionally conversion of disease burden. At present, accidents, cardiovascular diseases, cancers, and mental disorders are the highest rank in burden of disease. Rising of health costs, non-coverage of major costs by insurance, incomplete coverage of Primary Health Care (PHC) networks in urban areas, lack of referral system and fragmentation of policy making, are some important challenges of the health system in Iran(Rostamigooran, et al., 2013). Currently the health system of Iran has two main characteristics, firstly, it is network based and secondly, the health services providing system has been integrated into educational system(Rajabi, Esmailzadeh, Rostamigooran, & Majdzadeh, 2013). Also Iran, as a developing country with its idiosyncratic various geographical and climatic conditions has led to the emergence of geographically diverse landscapes over time passing through ups and downs; consequently, different parts of the country have not undergone similar development advantages, leading to inequality in the enjoyment of the blessings of the development and distribution of facilities and services (Ghanbari, 2011). Hence, it is required to define the terms of access to health services and then develop a comprehensive program to fix this problem. This study was conducted using TOPSIS technique to assess the health services access across the Markazi province.

## 2. Methods

This was a cross-sectional study. The sample included all 10 towns of Markazi province that is located almost in the center of Iran that limited to the provinces of Tehran and Qazvin from the north, to Hamadan from the west, to Lorestan and Esfahan from the south, and to Qom from the east. The city of Arak is the center of this province. Its population is estimated at 1325655 million that includes the townships of Arak (599634), Ashtian (17105), Delijan (48986), Khomein (107368), Komijan (39340), Mahallat (53381), Saveh (259030), Shazand (117746), Tafresh (25912) and Zarandiyeh (57153). According to the statistics, the average annual growth rate of the population of Markazi is 0.91 that have been calculated based on the data of 2011 and its population percentage is 1.88. Markazi province has a unique position in the country because of its fertile lands, and the production of some agricultural and livestock products. It can be said that approximately 50% of the people in Markazi province live in rural areas and their main occupation is farming and animal husbandry.

The numbers of 15 health indices were selected based on their availability at 2011 National Statistics Center’s annual report that included some indicators of availability of healthcare human and physical resources as follows: a) Number of laboratories per 1000 people, b) Number of rural active health house workers per1000 people, c) Number of general practitioners per 1000 people, d) Number of specialists per 1000 people, e) Number of paramedics per 1000 people, f) Number of active inpatient beds per 1000 people, g) Number of rural active health houses per 1000 people, h) Number of pharmacies to 1000 people, i) Number of pharmacologists per 10000 people, j) Number of dentists per 1000 people, k) Number of rural active health centers per 1000 people, l) Number of urban health centers per 1000 people, m) Number of radiology centers per 1000 people, n) Number of rehabilitation centers to 1000 people, o) Number of active treatment centers per 1000 people.

To evaluate and determine the development level, numerous quantitative methods and techniques are available which depending the validity and credibility of the existing information and the skills of local authorities are employed to plan, organize and evaluate past information (Badri & Akbarian Ronizi, 2007). One method of grading the areas according to facility distribution is the TOPSIS method. In the present study, indices are weighted based on Shannon entropy techniques, having been used as inputs to the TOPSIS algorithm. When data is delineated in a decision-making matrix and the decision maker wishes to compare the index weight with regard to such data, the Shannon entropy technique can be employed for index weighting. The method is based on the fact that the greater the distribution of values of an index, the more significant the index will be regarded (Mohammadi & Izadi, 2012).

Shannon entropy method included the following steps (Zyaree, Mohamadi, & Atar, 2012):

First, the raw data matrix was normalized according to the formula:


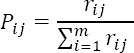


“*Pij*” is the normalized value of the index “*j*” in the “*i-^th^*” rank; “*r_ij_*” is the initial index value; and “*m*” is the number of options available to the ranking. Then “*E_j_*” (entropy per index) of “*P_ij_*”, for each index was calculated:


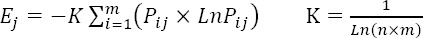


*“n”* is the number of variables and “*m*” the number of places which are compared with. Accordingly, the degree of uncertainty or standard deviation (d_j_) for each of the indices is obtained:


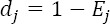


Finally, the weight of each indicator (W_j_) is calculated as follows:


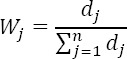


Nowadays, TOPSIS technique has gained significance as one of the multi-criteria decision-making techniques to rank the different concepts of various branches of science, the most important reason being it’s clear, mathematical logic and the practical problems associated with it. Moreover, the multiplicity of the criteria of the units to be compared has caused problems in decision-making techniques by the majority of techniques. Fortunately, this is not a problem in TOPSIS technique. Finally, the compensational property of these techniques aiming to provide reasonable rating appropriate to the perception of experts, justifies the employment of these powerful techniques (Zyaree, Zanjirchee, & Sorkhkamal, 2010). TOPSIS is done in the following steps (Zyaree et al., 2012):

First, the maximum 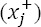 and minimum 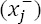 values of each index are identified. Then, using the following equation, normalization takes place. If the positive and negative indices are intended to be combined reversing the negative aspects into positive aspects should be done as follows:


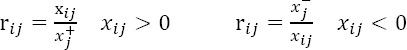


The standard weighted matrix based on the following equation is obtained:


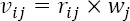


The positive ideal and negative ideal solutions for each of the indices are determined by the following procedure:


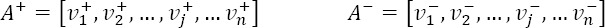


The positive ideal index is equal to its maximum, and the negative ideal in every index, the index is equal to the minimum. Distance of each option compared with ideals of positive and negative, are as follows (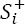, 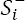):

Distance option *i* from the positive ideal:


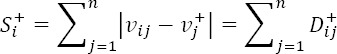


Distance option *i* from the negative ideal:


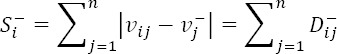


For calculation of relative closeness of each alternative to the ideal we should combine the values of 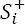 and 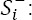



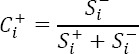


The ranking is done based on the decreasing values of 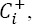 means that the highest 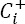 is considered as the most developed, and the lowest 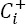 as most undeveloped.

## 3. Result

The aim of this study is to provide a clear vision from the status of Markazi cities in terms of access to health services. First, using Shannon entropy techniques, weight and ranking indices for determining the degree of development of Markazi cities is derived (Table 1).

**Table 1:** Titles, ranking and weighing indices to determine the degree of development

Rank	Indices	Weight
1	Number of urban health centers per 1000 people	0.0806
2	Number of rehabilitation centers to 1000 people	0.0784
3	Number of pharmacy to 1000 people	0.0731
4	Number of radiology centers per 1000 people	0.0698
5	Number of active treatment centers per 1000 people	0.0681
6	Number of specialist per 1000 people	0.0676
7	Number of laboratory per 1000 people	0.0662
8	Number of general practitioner per 1000 people	0.0653
9	Number of rural active health center per 1000 people	0.0643
10	Number of dentist per 1000 people	0.0642
11	Number of active beds of treatment centers per 1000 people	0.0636
12	Number of pharmacologist per 10000 people	0.0610
13	Number of paramedical per 1000 people	0.0601
14	Number of rural active health house workers per 1000 people	0.0590
15	Number of rural active health house per 1000 people	0.0584

As Table 1 shows, among the 15 indices of health, the number of urban health centers per 1000 people with weight of 0.0806 and number of rural active health house per 1000 people with weight of 0.0584 had the highest (1^th^) and lowest (15^th^) ranks respectively.

Using TOPSIS technique, Markazi towns were compared in terms of access to health services. In order to define a priority measure and for a better understanding of the status to health services access in Markazi, 10 towns were assessed and ranked into three categories (Table 2).

**Table 2:** Markazi cities ranking in the degree of development

Rank	City name	Coefficient of development	Degree of development
1	Ashtian	0.802	Developed
2	Mahallat	0.227	
3	Khomein	0.190	Developing & Underdeveloped
4	Zarandiyeh	0.184	
5	Arak	0.152	
6	Delijan	0.150	
7	Komijan	0.147	
8	Tafresh	0.130	
9	Saveh	0.122	
10	Shazand	0.077	

The results show that the highest and lowest degrees of development based on TOPSIS technique in Markazi province, belonged to Ashtian (0.802) and Shazand (0.077) respectively. Results indicate that in terms of access to health services, Ashtian can be regarded as developed, while Mahallat, Khomein, Zarandiyeh, Arak, Delijan, Komijan, Tafresh, Saveh and Shazand are categorized as developing and underdeveloped. The findings show a large gap between Markazi towns in terms of access to health services.

## 4. Discussion

Iran as a developing country has a lot of problems and contrasts in terms of various development indicators. One of the indicators of the development that has a disproportionate geographical distribution among the cities is health and care indicators. With regard to the awareness development situation in a region, it needs to check the structure, process and outcome indicators simultaneously. But in present study due to limitations in data availability, it was only analyzed access to health care facilities in Markazi province, Thus, we study the development of health services in the province to determine measure of differences in the level of development between them and Finally, providing guidance to officials and policy makers for allocating resources in the most deprived and undeveloped areas and pay more attention to them.

The results of this study showed that there is a large gap between health indicators in different cities of Markazi province. The one city (Ashtian) can be classified as developed cities, nine other cities (Mahallat, Khomein, Zarandiyeh, Arak, Delijan, Komijan, Tafresh, Saveh and Shazand) can be classified as in developing and underdeveloped cities level. Respectively, Ashtian and Shazand obtained the highest and lowest enjoyment rate the indicators. In national (Anjomshoa et al., 2014; Mousavi et al., 2013; Sabermahani, Barouni, Seyedin, & Aryankhesal, 2013; Sadeghifar et al., 2014; Taghvaei & Shahivandi, 2011) and international studies(Asanin & Wilson, 2008; Boutayeb & Helmert, 2011; Fang et al., 2010; Horev, Pesis-Katz, & Mukamel, 2004; Kreng & Yang, 2011; Peters et al., 2008; Pong, Desmeules, & Lagace, 2009; Theodorakis, Mantzavinis, Rrumbullaku, Lionis, & Trell, 2006) similar results have been obtained in terms of enjoyment of health indicators.

It is worth noting that having high enjoyment level of health indices in one city cannot be a logical reason of high quality level accessibility of health care services. In other words, the cities which have located in developing and undeveloped level are faced with health care facilities quantitatively while the quality of service in cities extremely depends on methods of facilities organizing, population characteristic of service receiver and other different factors.

The gap in terms of access to health care facilities has been observed both among countries and different regions of a country. The existence of a balanced development in certain aspects (such as cultural, social and economic development and etc.) and different geographical locations is a necessity. Planners and policymakers should focus their efforts on finding the reason of development gaps and distinctions and overcoming the related problems. In order to reduce the existing heath gap between cities and the even distribution of health care services, it is crucial to develop a general and comprehensive plan for covering the macro–scale and top-down program with a focus intention of reaching a micro and local plan in a micro-scale.

## 5. Conclusion

Based on the results, there is a large gap and distinction between Markazi province cities in terms of access to health care facilities. The most important factor in the unequal distribution of health services in different areas is policies and programs that lead to a concentration of services in big cities. This causes some difficulties to access health services in different areas so that big cities can attract hospital facilities, pharmacies, advanced rehabilitation centers and human resources specialists due to financial and investment attractiveness. Therefore, policy makers and administrators should decrease the distinction and gap of enjoyment of health care facilities to attain the fair and balanced health situation in accordance with the state of development and planning of cities based on gathered facts. This study refers to importance of following major achievements for planners and policy makers at national and local level: To aid the understanding of the current situation in the province from access to health care, helping to better decisions to improve less-developed cities and according to these cities to develop short-and long-term plan to reduce the gap in development.

## 6. Limitations

One of the limitations of this study could be effect on the results is analyzing of health indicators in only one province of Iran. Therefore, it is imperative that further research should be conducted in other provinces. It is noted that this study only examined the health indicators, and therefore it is recommended that other indicators of development should be considered in future studies.
